# Machine learning-based prediction of intraoperative blood transfusion in major surgery: exploiting clinical variables and systemic inflammatory indices

**DOI:** 10.1080/07853890.2026.2707807

**Published:** 2026-08-10

**Authors:** Laura Verzellesi, Lucia Merolle, Marco Bertolini, Valeria Trojani, Davide Schiroli, Andrea Nitrosi, Erminia Di Bartolomeo, Margherita Genitoni, Mauro Iori, Roberto Baricchi, Chiara Marraccini

**Affiliations:** ^a^ Medical Physics Unit, AUSL-IRCCS di Reggio Emilia, Reggio Emilia, Italy; ^b^ Enzo Ferrari Department of Engineering, University of Modena and Reggio Emilia, Modena, Italy; ^c^ Department of Computer Sciences, University of Pisa, Pisa, Italy; ^d^ Transfusion Medicine Unit, AUSL-IRCCS di Reggio Emilia, Reggio Emilia, Italy; ^e^ Clinical and Experimental Medicine PhD Program, University of Modena and Reggio Emilia, Modena, Italy

**Keywords:** Machine learning, CatBoost model, intraoperative transfusion, patient blood management, major surgery, oncological surgery, inflammatory ratios, monocyte-to lymphocyte ratio, platelet-to-lymphocyte ratio, neutrophil-to lymphocyte ratio

## Abstract

**Background:**

Intraoperative blood transfusion is common in major surgery. Predicting transfusion risk may improve perioperative management, optimize blood use, and enhance surgical planning. Machine Learning (ML) offers promising tools for individualized risk prediction.

**Aim:**

To develop and validate an ML model predicting intraoperative transfusion risk in major surgery using clinical variables and inflammatory indices.

**Material and methods:**

1858 adult patients who underwent major surgical procedures at AUSL-IRCCS di Reggio Emilia between September 2021 and December 2023 were retrospectively analyzed. The dataset was splitted into training (60%), internal validation (18%) and internal test (22%) sets. The primary outcome was the intraoperative red blood cell transfusion. Thirty-three candidate variables (29 clinical and laboratory parameters and 4 calculated indices) were evaluated. Highly correlated variables were excluded and key predictors were identified using CatBoost. Predictors distributions were compared by transfusion status and oncological diagnosis. Model performance was assessed by AUC, sensitivity, specificity, NPV, PPV, and F1-score. SHAP graph were used to interpret feature contributions.

**Results:**

The CatBoost model demonstrated strong predictive performance (AUC 0.88 training, 0.80 test). and a high NPV (0.89 training, 0.87 test), reliably identifyinglow-risk patients. Key predictors included type of surgery, preoperative haemoglobin, age, BMI, MCV RDW, PT and inflammatory indices such as MLR, PLR, NLR.

**Conclusions:**

This ML-driven model accurately predicts intraoperative transfusion risk and identifies clinical and laboratory predictors, supporting perioperative management and more efficient resource allocation.

## Introduction

Major surgery is often accompanied by perioperative blood loss, placing many patients at increased risk of red blood cell (RBC) transfusion. The likelihood of being transfused may depend on several factors, including the type of surgery (e.g. cardiovascular, orthopaedics, or cancer resection) [1–3] or pre-existing patient’s conditions (e.g. chronic kidney disease, inflammatory disorders, or anaemia) [4–7].

In the last decades, Patient Blood Management (PBM) has emerged as an evidence-based, multidisciplinary approach designed to optimize the care of patients who may require transfusions [8–10]. A primary focus of PBM is the preoperative identification and correction of bleeding risk factors, which are also key determinants of morbidity and mortality [3,9,11,12]. Preoperative anaemia affects 20–40% of surgical patients, peaking at over 60% in colorectal surgery cases [13]. Large observational studies have demonstrated that preoperative anaemia, even mild, is an independent predictor of postoperative complications and mortality, with a clear severity–response relationship [14–16]. Therefore, early detection and optimization of anaemia is crucial for patient outcomes, which highlights the importance of PBM approaches.

While RBC transfusion is potentially life-saving, its use is not without risk, and minimizing unnecessary transfusions through careful patient assessment and appropriate thresholds is essential. Accumulating evidence, indeed, has linked perioperative transfusion to increased morbidity and mortality [17], including higher rates of infection, thromboembolic events, acute lung injury, renal dysfunction, and long-term adverse outcomes [13,18–20].

Recently, both non-transfusion strategies [9,21] and approaches aimed at improving transfusion appropriateness [3,12] have proven effective in reducing exposure to allogeneic blood products, improving postoperative recovery, and optimizing resource utilization. These strategies include correcting preoperative anaemia with intravenous iron supplementation, managing nutritional deficiencies, selectively using erythropoiesis-stimulating agents, and implementing point-of-care continuous monitoring of haemoglobin (Hb) levels within PBM programs. In this context, there is a need to improve the pre-operative stratification of patients in order to accurately identify individuals who can avoid blood transfusions through the preventive correction of anaemia.

Beyond conventional clinical and laboratory variables, increasing attention has recently been directed toward systemic inflammatory biomarkers derived from routine blood counts, including the neutrophil-to-lymphocyte ratio (NLR), monocyte-to-lymphocyte ratio (MLR), platelet-to-lymphocyte ratio (PLR), and eosinophil-to-lymphocyte ratio (ELR). These lymphocyte-based inflammatory ratios have emerged as accessible indicators of the systemic inflammatory response and have shown prognostic value in several surgical and oncological settings [22–24]. Chronic inflammation is a well-recognized driver of impaired erythropoiesis through reduced erythropoietin production, iron dysregulation, and suppression of red blood cell production [25–27], particularly in cancer-related anaemia. Nevertheless, despite their biological plausibility and increasing clinical interest, the contribution of these inflammatory ratios to perioperative transfusion risk remains poorly understood, and, to our knowledge, they have not yet been systematically incorporated into machine learning models for transfusion prediction [28,29].

Machine learning (ML) can help develop predictive tools to support clinicians in making individualized, evidence-based transfusion decisions. These tools can enhance perioperative safety and promote responsible stewardship of blood resources within the PBM paradigm [30,31]. Enabling the learning of complex, nonlinear patterns from large datasets that exceed the capabilities of traditional statistical methods, ML-based predictive models can provide accurate and individualized estimates of transfusion risk, thereby guiding targeted preoperative optimization and supporting evidence-based clinical decision-making [32].

Despite the adoption of ML approaches has the potential to fundamentally reshape transfusion practices and improve patient outcomes [33], most existing transfusion prediction models rely on conventional logistic regression, focus on single surgical fields, or lack generalizability across diverse patient populations [34,35]. Given these limitations, there is an unmet need for robust, data-driven tools that can accurately predict intraoperative transfusion across a wide range of surgical patients. In this study, we developed and validated a predictive model using CatBoost [36], a gradient boosting algorithm, to estimate the risk of receiving intraoperative RBC transfusion during major surgery.

## Materials and methods

### Study design and population

This retrospective cohort study included all adult patients (>18 years) who underwent major surgery at the AUSL-IRCCS di Reggio Emilia (Italy) between September 2021 and December 2023. The study was approved by Area Vasta Emilia Nord Ethical Committee, the Ethics Committee responsible for reviewing research conducted at the AUSL – IRCCS di Reggio Emilia, Italy. Ethical approval was granted on June 10, 2025 (protocol No. 2025/0080971). Given the retrospective nature of the study and the use of previously collected clinical data, the requirement for informed consent was waived when patients were deceased or not contactable, in accordance with national regulations. The study was conducted in accordance with the Declaration of Helsinki and relevant national regulations. Data extraction from informatics database and variable collection were carried out between August 1, 2025 and September 30, 2025. Subsequently, data cleaning, processing, and statistical analyses were performed between October 1, 2025 and January 31, 2026.

### Variable selection and definitions

A total of 33 demographic, clinical and laboratory variables were selected for training the algorithm, informed by a comprehensive review of the literature, clinical expertise, and multidisciplinary discussion. Of these, 29 were extracted from three electronic medical record systems and included clinical characteristics, diagnoses, surgical type, tumour stage and laboratory parameters, whereas four variables were calculated as inflammatory ratios: the platelet-to-lymphocyte ratio (PLR), the monocyte-to-lymphocyte ratio (MLR), the neutrophil-to-lymphocyte ratio (NLR), and the eosinophil-to-lymphocyte ratio (ELR). Demographic information included age, sex, and body mass index (BMI). For preoperative laboratory variables with multiple available measurements, the variable closest to the fifteenth day prior to the scheduled date of the procedure was selected. The type of surgery was defined according to ICD-9 categories [37]. Only procedural codes were included, excluding diagnostic codes and grouped by anatomical district (see Supplementary Table 1).

The primary outcome was intraoperative RBC transfusion, defined as the administration of ≥1 unit of packed RBC during surgery. For binary classification, transfusion was determined as administration of ≥1 unit (class 1), while no transfusion was defined as 0 units (class 0).

### Modelling strategy

**Figure 1.**
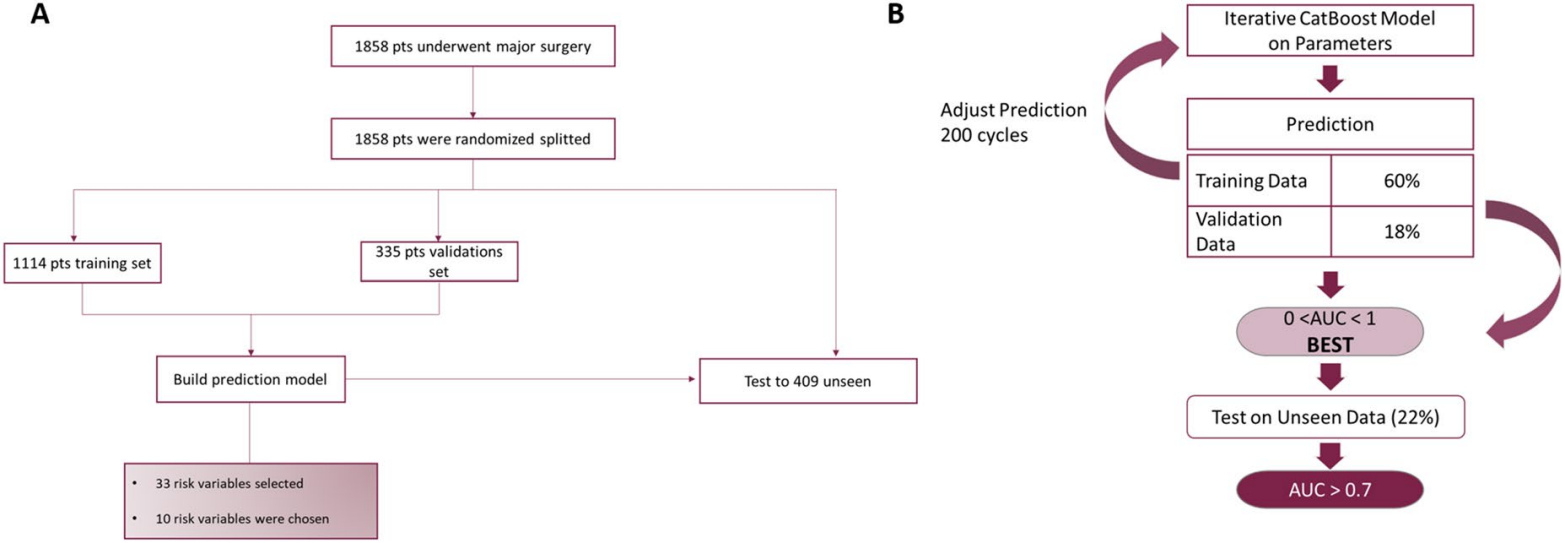
Flowchart of the study (A) and modelling strategy (B).

The dataset was randomly divided using stratified sampling on the binary target to preserve class prevalence. Specifically, a first split allocated 60% of the records to the training set and 40% to a provisional holdout set. The provisional holdout set was then split into the validation and test subsets with stratification, resulting in approximately 60%, 18% and 22% partitions for training, validation and test, respectively. Figure 1 shows a synopsis of the flowchart of the study (panel A) and the modelling strategy (panel B). Briefly, the 1858 patients recruited were randomly assigned to the training (*n* = 1114), validation (*n* = 385) and test set (*n* = 409). The CatBoost model was trained on the training set, while model tuning and performance monitoring were conducted using the validation set.

Catboost, an algorithm for gradient boosting on decision trees [36], can handle both missing data and categorical variables natively, without the need for preliminary imputation or encoding. During tree construction, CatBoost automatically learns the optimal split direction for missing values, thereby including patients with incomplete information in the model while minimizing data loss and potential imputation-related bias.

Numerical predictors were scaled using min-max normalization [38]. The scaler was fit on the training set only and subsequently applied to validation and test sets to prevent data leakage. Categorical features were not scaled. Feature redundancy among numerical variables was assessed using a correlation-based filter: for any pair of features with a Pearson correlation coefficient greater than 0.80, only one feature from the pair was retained for further analysis (Figure 1). CatBoost was trained to predict intraoperative transfusion risk. To mitigate class imbalance, automatic class weighting was enabled (auto_class_weights = ‘Balanced’).

Hyperparameters were optimized using Hyperopt [39]. The optimization target was the Area under the ROC Curve (AUC) computed on the validation set. The search was run for 200 evaluations (max_evals = 200) across the following space: number of iterations {140,150,160,180,200}, tree depth {4,6,8,10,12,14,16}, learning rate (log-uniform 0.01–0.2), L2 regularization (uniform 25–30), subsampling (uniform 0.5–0.75), border count {32,64,128,254}, random strength (uniform 20–35), and bagging temperature (uniform 10–20).

After selecting the best hyperparameters, feature importance was extracted, and the top 10 predictors were selected. The final model was refit using the best hyperparameters and the selected features.

### Statistical analysis

Quantitative data were expressed as mean ± standard deviation (SD) or median and interquartile range (min-max) as appropriate, and qualitative data were expressed as frequency (percentage). Student’s t-test was utilised to compare quantitative variables normally distributed. The Mann-Whitney U test was used to compare non-Gaussian distributed variables.

Discrimination power was quantified using the AUC. Classification metrics were computed from the confusion matrix at a probability threshold of 0.5, including sensitivity (SENS), specificity (SPEC), negative predictive value (NPV), positive predictive value (PPV), and F1-score [40,41]. The threshold of 0.5 was selected as a conventional default for binary classification to provide a neutral and reproducible evaluation of model performance. In future clinical contexts, a threshold optimization based on clinical priorities (e.g. maximizing sensitivity or NPV) may help to further evidence clinical utility and should be explored in future studies. To quantify metric uncertainty, a stratified bootstrap procedure was implemented (1,000 bootstrap samples), resampling positive and negative cases separately to preserve class prevalence, and reporting percentile confidence intervals (5th–95th percentiles). Calibration metrics are reported in Supplementary information (Figure S1, Supplementary Table 2).

Model explainability was assessed using SHAP (Shapley Additive Explanation) [33], a Python-based tool that interprets complex machine learning models by quantifying how much each feature contributes to the model’s predictions. A higher SHAP score indicates a stronger association with the likelihood of receiving an intraoperative transfusion. Global feature impact was summarized using SHAP summary plots, and feature-specific effects were explored *via* SHAP dependence plots, including analyses stratified by surgical category.

All analyses were performed using Python (Version 3.12.4) and R (Version 3.6). *p* < 0.05 was considered statistically significant.

## Results

### Clinical characteristics of the study population

The study comprised 1,858 adult patients who underwent major surgery between September 2021 and December 2023. Among these, 958 were diagnosed with cancer. Table 1 shows a comprehensive overview of the baseline characteristics, grouped by area of surgery. The mean age of the total population was 70.3 ± 13.5 years, with 835 females (70.5 ± 14.6 years) and 1023 males (69.9 ± 12.6 years). A total of 664 patients received at least one RBC transfusion during surgery. Of these patients, 317 were female. Overall, 38% of the female patients (317 out of 835) received intraoperative transfusions, compared to 34% of the male patients (347 out of 1,023) (see Supplementary Table 3 for details on the main features of females and males). The distribution across surgical groups revealed substantial heterogeneity in both sex and age (Table 1).

**Table 1.** Age distribution of the study population by type of surgery and sex.

Type of surgery	Females (*n* = 835)					Males (*n* = 1023)				
	n	mean	SD	median	min-max	n	mean	SD	median	min-max
**Abdominal surgery**	26	59.9	12.2	65	33–80	2	49.5	23.3	49	33–66
**Digestive surgery**	174	66.7	13.3	67	25–90	244	65.7	13.3	67	18–88
**Kidney**	47	67.5	10.7	66	43–87	113	67.5	12.0	69	32–87
**Endocrine surgery**	25	51.8	11.0	52	33–75	15	54.9	12.2	56	32–76
**Gynecologic surgery**	69	64.3	12.6	65	32–90	0				
**Prostate surgery**	0					83	68.4	7.4	68	47–89
**Lung surgery**	19	66.7	10.0	70	48–79	41	67.4	8.8	71	43–78
**Vascular surgery**	15	74.7	10.6	79	45–86	74	74.4	8.5	76	53–90
**Bladder surgery**	10	74.7	12.7	76	42–89	24	76.2	3.6	76	68–82
**Urinary tract surgery**	78	61.8	17.6	61	20–91	188	71.9	11.2	74	31–94
**Liver surgery**	8	73.1	10.3	73	60–84	13	71.6	6.3	74	58–81
**Orthopedic surgery**	356	78.6	11.3	81	18–98	216	74.1	14.6	75	18–98
**Trauma**	1	63		63		0				
**Other**	3	49.0	9.6	45	42–60	5	66.0	16.2	68	42–82
**Not specified**	4	71.8	12.3	75	55–82	5	62.2	6.7	66	54–69

Values are expressed as number of patients (n), mean, standard deviation of the mean (SD), median with minimum–maximum values (min-max).

The largest subgroup, comprising 572 patients, underwent orthopaedic surgery. Within this group, female patients were older than males (78.6 ± 11.3 *vs.* 74.1 ± 14.6 years). According to the comparative analysis, digestive system surgery (*n* = 418) showed comparable age distribution across sexes, whereas kidney and urinary tract surgery (*n* = 426) exhibited a pronounced male predominance.

### Modelling strategy

**Figure 2.**
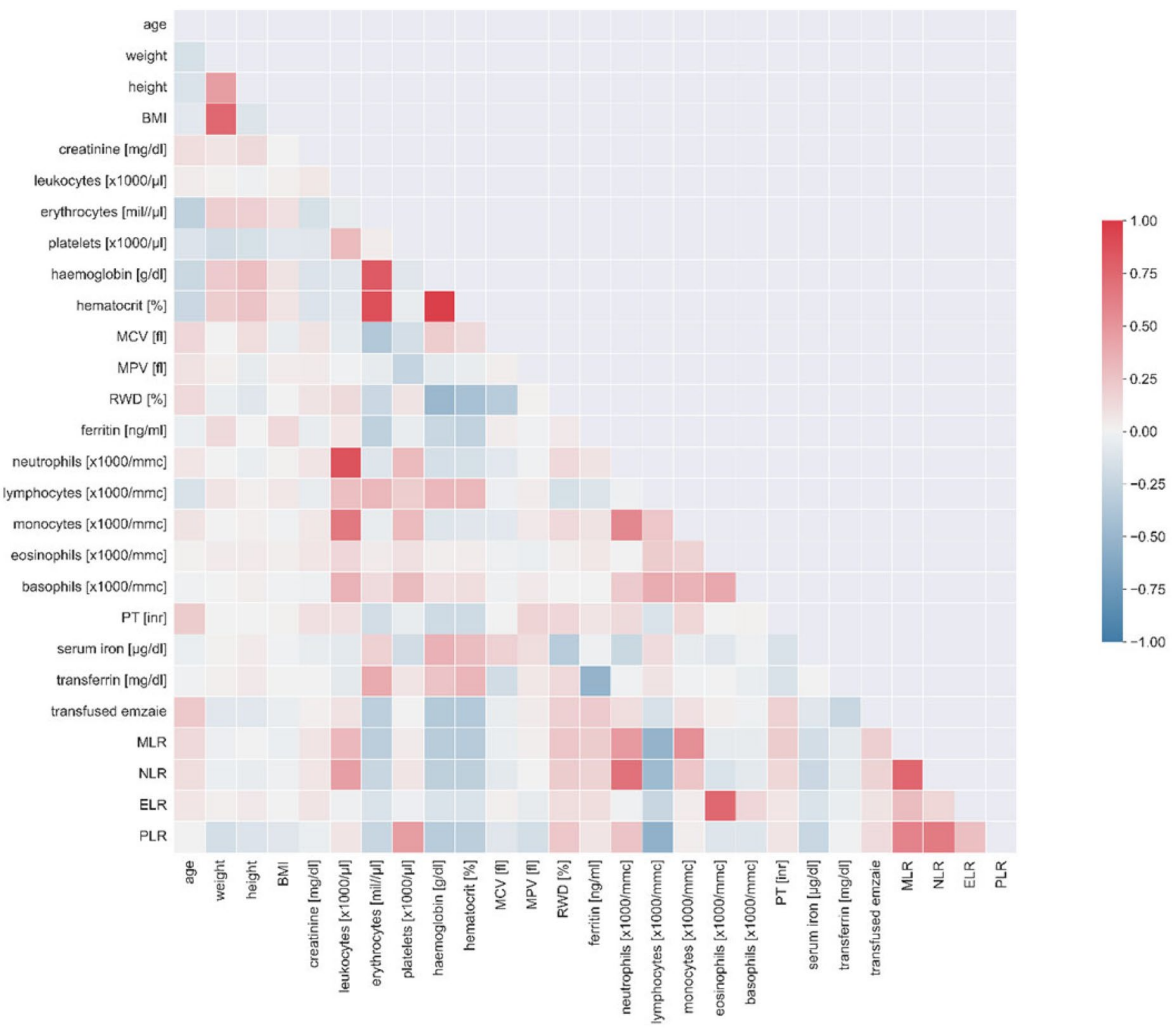
Heatmap describing the correlation matrix among the numeric variables collected for each patient. Relationships among the variables were assessed by calculating the Pearson correlation coefficients. Couples of variables were deemed to be correlated when R was greater than 0.8, and only one was selected for CatBoost training (specifically, total leucocytes, total RBC and haematocrit have been removed from the model training).

A total of 33 candidate variables were initially considered, including 29 directly extracted clinical and laboratory parameters and 4 derived indices.To avoid feature redundancy among the variables used to train CatBoost, the relationships among them were assessed by calculating the Pearson correlation coefficients, which were then visualised in a heatmap. Figure 2 shows the correlation matrix used to identify highly correlated variables: the absolute values of the correlation coefficients ranged from −1 to 1, with higher values indicating stronger correlations. Positive coefficients are indicative of positive correlations, whilst negative coefficients are suggestive of inverse relationships. For instance, a negative correlation (R = −0.49) was identified between haemoglobin and Red blood cell Distribution Width (RDW). Couples of variables were deemed to be correlated when R was greater than 0.8, and only one was selected for CatBoost training (specifically, total leucocytes, total RBC and haematocrit have been removed). Therefore, the predictive model was built on 26 variables and 4 calculated ratios (i.e. PLR, MLR, NLR and ELR).

### Model performance evaluation and key features

**Figure 3.**
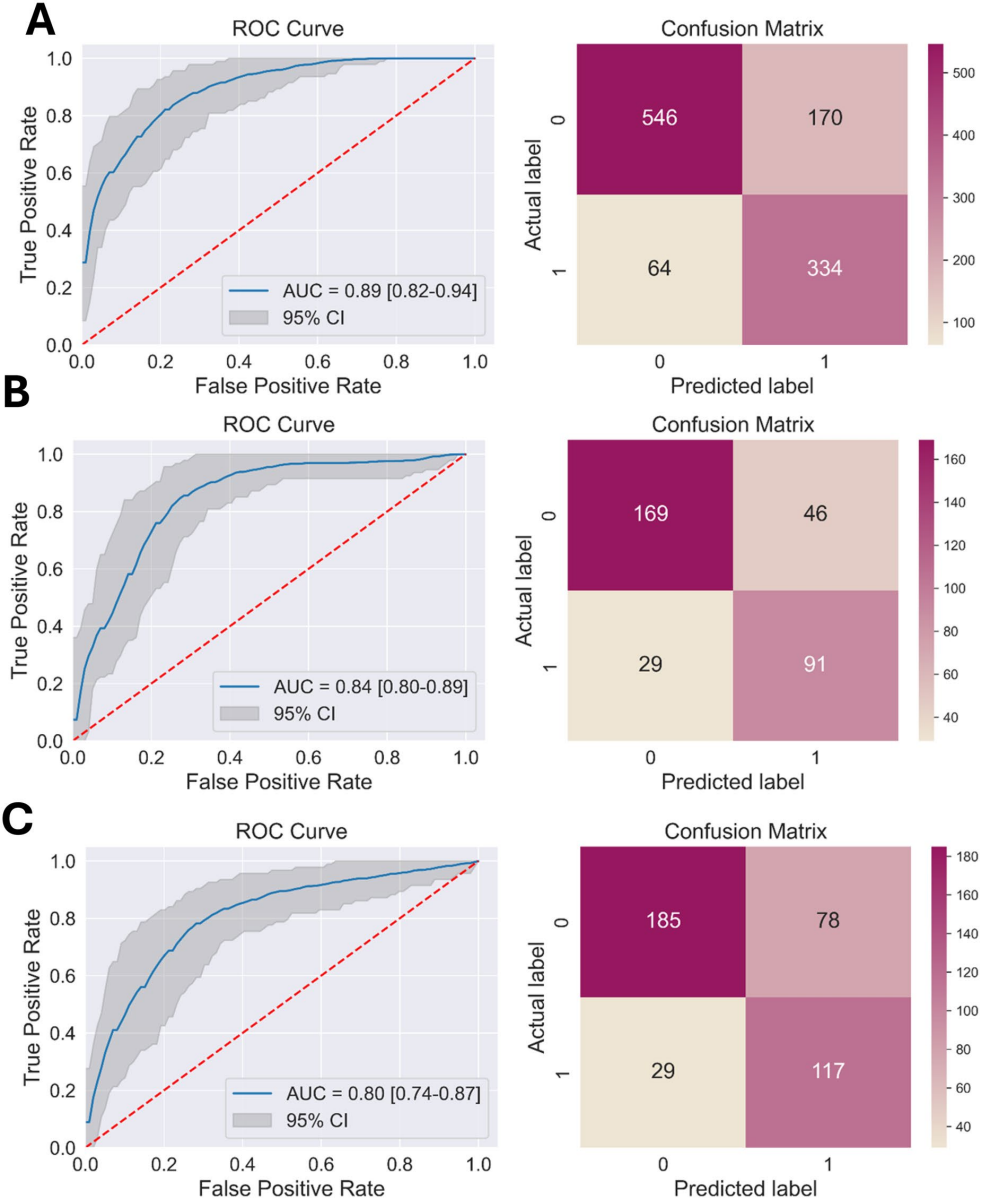
Model’s performance. AUCs and confusion matrices for training (A), validating (B), and test (C) dataset.

With its top 10 selected predictors, the model demonstrated high discriminative performance across the three datasets. Full performance metrics, including AUCs, positive predictive value, specificity, sensitivity, and class-wise F1 scores with corresponding 95% confidence intervals, are reported in Table 2. In particular, the AUC was 0.885 (95% CI: 0.824–0.935) in the training set (Table 2 and Figure 3A) and 0.841 (0.797–0.885) in the validation set (Figure 3B). Final model performance was assessed on the independent test set, consisting of previously unseen data, yielding an AUC of 0.805 (0.739–0.872) (Figure 3C). Overall, the model maintained a high negative predictive value and good sensitivity for identifying patients requiring intraoperative RBC transfusion.

**Table 2.** Model’s performance metrics with corresponding 95% CIs in square brackets.

Set	AUC	NPV	PPV	SPEC	SENS	F1 (class = 0)	F1 (class = 1)
**Train**	0.885	0.894	0.662	0.759	0.838	0.82	0.739
	[0.824–0.935]	[0.833–0.955]	[0.596–0.737]	[0.690–0.822]	[0.723–0.936]	[0.772–0.873]	[0.667–0.811]
**Validation**	0.841	0.852	0.66	0.78	0.757	0.814	0.704
	[0.797–0.885]	[0.810–0.901]	[0.590–0.725]	[0.714–0.845]	[0.660–0.851]	[0.765–0.854]	[0.646–0.767]
**Test**	0.805	0.868	0.599	0.695	0.81	0.771	0.688
	[0.739–0.872]	[0.808–0.927]	[0.523–0.672]	[0.618–0.774]	[0.702–0.895]	[0.710–0.829]	[0.605–0.759]

AUC = Area Under the Curve; NPV = Negative Predictive Value; SPEC = Specificity; SENS = Sensitivity; F1 = F1-score (harmonic mean of precision and recall).

**Figure 4.**
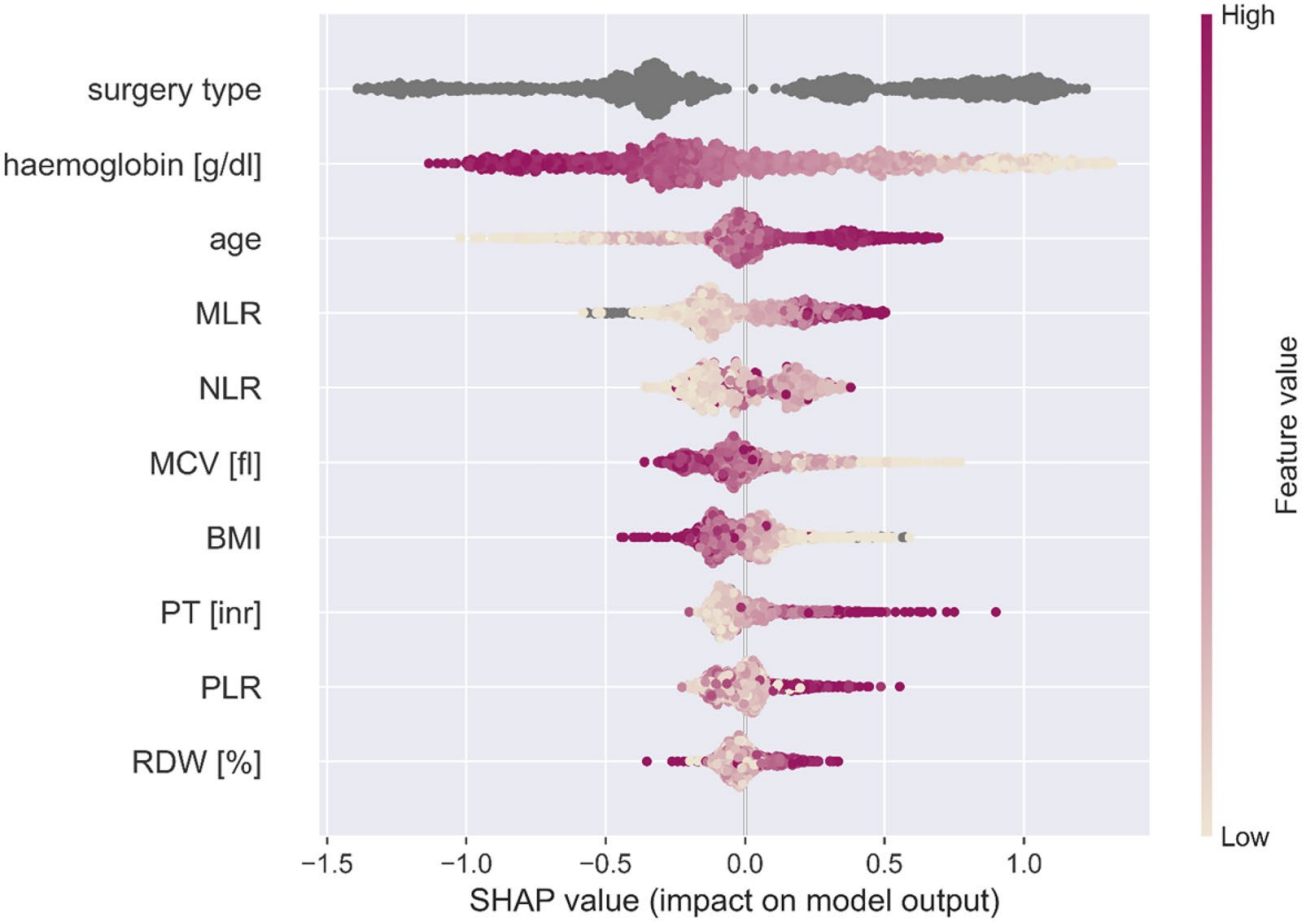
SHAP analysis of the CatBoost model. Each point represents a sample, and a larger area indicates that many samples are collected. The color on the right indicates the value of the feature; purple indicates that the feature value is high, and pink indicates that the feature value is low.

Figure 4 illustrates the SHAP summary plot. The features are arranged in accordance with their relative impact, with the most impactful predictors appearing at the top of the plot and the least impactful features appearing at the bottom. The colour coding denotes the feature value for each observation: dark purple indicates high values and light pink indicates low values. The type of surgery, which is a categorical feature, is grey-coloured. According to the SHAP, lowlevels of haemoglobin and advanced age are associated with an increased risk of intra-operative transfusion. Conversely, high MLR and NLR levels are associated with higher SHAP values evidencing an increased probability of being transfused during surgery.

**Figure 5.**
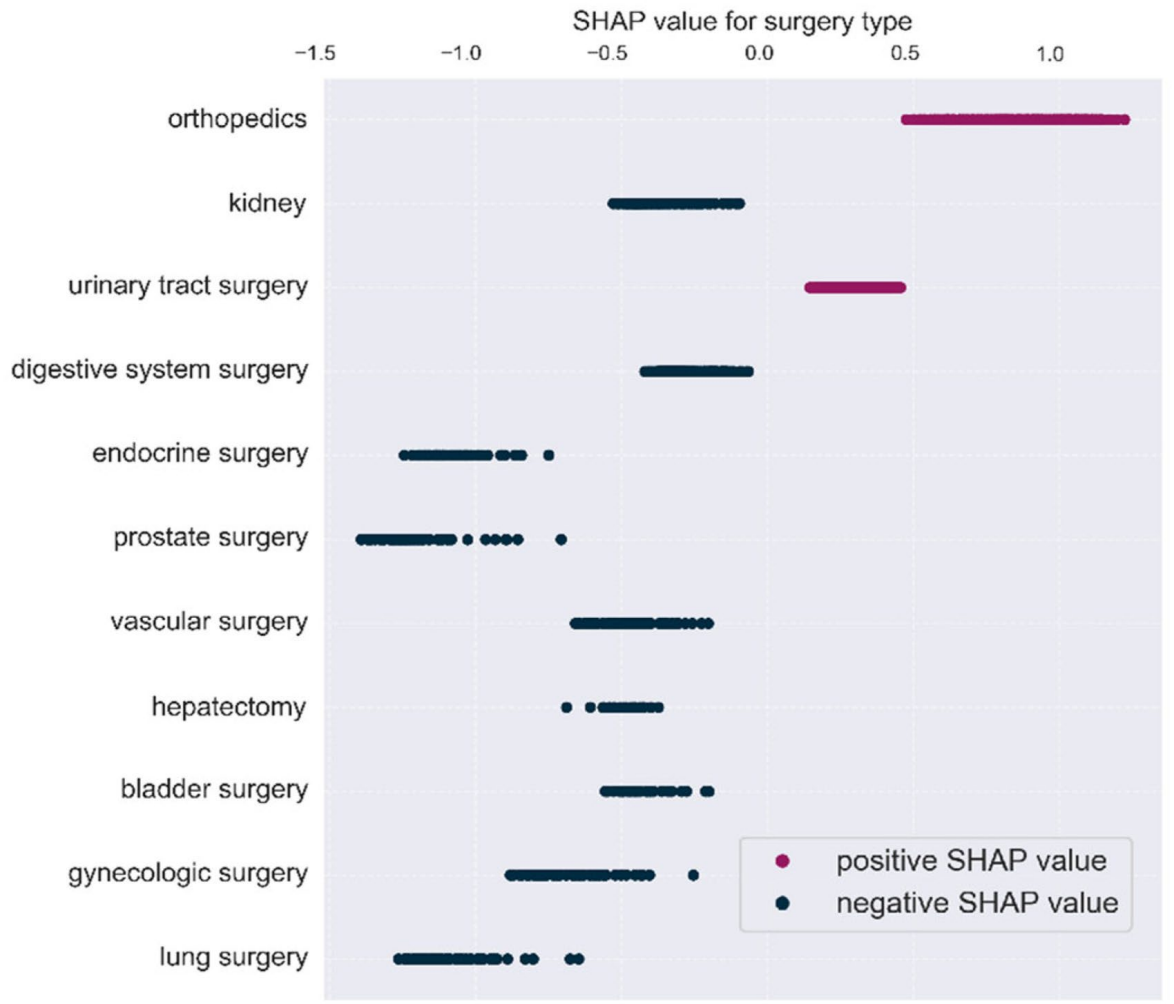
SHAP analysis of the CatBoost model stratifying for type of surgery.

Figure 5 shows in detail the corresponding dependency plot for the most common groups of surgical procedures found in our cohort of patients. The predicted risk of intraoperative transfusion is higher for all types of surgery associated with a positive SHAP value than for the model’s baseline. The highest positive mean SHAP values were revealed for orthopaedic and urinary tract surgeries, representing the strongest individual contributors to an increased predicted transfusion risk. Meanwhile, a negative value means the risk decreases. This was found for lung, gynaecological and bladder surgeries, suggesting they were predictive of a reduced risk of transfusion within the model’s framework.

The descriptive statistics for the selected predictive features were stratified by patients who did and did not received transfusions (Table 3). Among the 1,858 patients analysed, 664 (35.7%) received at least one unit of RBC during surgery. The transfused cohort had a significantly lower median pre-operative haemoglobin level (11.8 g/dL; range 7.0–16.7) than the non-transfused cohort (13.5 g/dL; range 6.4–18.8). Furthermore, patients who received a transfusion were markedly older, with a median age of 77 years (range 18–98) versus 70 years (range 18–96). Several other significant differences were also observed in the data. Firstly, there was a notable difference in BMI (median 25.2 vs. 26.1 kg/m^2^). Secondly, there was a significant difference in RDW (median 14.4% vs. 13.6%). Coagulation status, as measured by PT, was marginally higher in the transfused group (median 1.1 vs. 1.0). Additionally, elevated levels of MLR, NLR, and PLR were observed in patients requiring transfusion.

**Table 3.** Descriptive statistics of the variables identified by the predictive algorithm as significant features associated with the probability of intraoperative RBC transfusion.

Predictor	Not Transfused (*n* = 1194)					Transfused (*n* = 664)					
	n	mean	SD	median	min-max	n	mean	SD	median	min-max	p-value
**Haemoglobin (g/dL)**	1194	13.4	1.8	13.5	6.4–18.8	664	11.8	2.0	11.8	7.0–16.7	<0.001
**Age (years)**	1194	67.5	13.2	70	18–96	664	75.3	12.4	77	18–98	<0.001
**Body Mass Index (BMI, kg/m²)**	1187	27.0	7.5	26.1	15.9–53.6	652	25.9	5.2	25.2	15.5–67.2	<0.001
**Mean Corpuscular Volume (MCV, fL)**	1194	90.8	6.2	91.1	63.4–123.0	664	89.6	7.8	90.5	61.0–114.0	0.008
**Red blood cell Distribution Width (RDW, %)**	1190	14.1	2.0	13.6	11.1–28.6	661	15.0	2.3	14.4	11.8–25.2	<0.001
**Prothrombin time (PT, inr)**	1086	1.1	0.3	1.0	0.8–5.1	539	1.2	0.4	1.1	0.9–4.4	<0.001
**Monocyte-to-Lymphocyte Ratio (MLR)**	1168	0.4	0.2	0.2	0.1–2.6	650	0.4	0.3	0.4	0.0–2.8	<0.001
**Neutrophil-to-Lymphocyte Ratio (NLR)**	1168	2.7	2.1	2.4	0.5–32.4	650	3.8	2.6	4.2	0.4–47.7	<0.001
**Platelet-to-Lymphocyte Ratio (PLR)**	1167	149.0	129.0	94.3	10.5–1460.0	650	177.0	144.0	121.0	33.8–1529.0	<0.001

The table reports the distribution (number of patients n, median, min and max) of the key features, comparing patients who did not receive transfusion with those who received ≥1 unit. P-values were calculated according to the Wilcoxon-Mann-Whitney test.

### Subgroup analysis on cancer patients

Although this has yet to be confirmed, there is substantial concern that blood transfusions are independently associated with worse perioperative and oncological outcomes [42–44]. Blood transfusions may provide additional risk for cancer patients by inducing transfusion-related immunomodulation, which can impair anti-tumour immune surveillance and promote infection, tumour progression, and adverse oncologic outcomes [45]. In this context, particular attention should be paid to the management of cancer patients undergoing surgery.

To determine whether the predictors identified by the model exhibited distinct patterns or significant differences in oncologic patients compared with non-oncologic patients, we performed a subgroup analysis based on oncologic diagnosis. As reported in Table 4, only three out of the 10 predictors emerged as statistically significant between oncological (*n* = 958) and non-oncological patients (*n* = 900). Patients diagnosed with cancer exhibited a higher median pre-operative haemoglobin level (13.20 g/dL vs 12.90 g/dL) and a lower median age (70 years vs. 75 years) compared to non-cancer patients. This data was partly expected, given the large number of elderly patients undergoing orthopaedic surgery (such as hip arthroplasty) in the non-oncologic cohort. Besides a lower median PT (1.01 vs. 1.04 inr), no other significant difference was observed between oncological and non-oncological groups (see Table 4), thus strengthening the generalisability of the predictive model.

**Table 4.** Descriptive statistics of the variables identified by the predictive algorithm as relevant features, stratified by oncological and non-oncological populations.

Predictor	Non oncologic (*n* = 900) transfused *n* = 330					Oncologic (*n* = 958) transfused *n* = 334					
	mean	median	SD	min	max	mean	median	SD	min	max	p-value
**Age (years)**	72.3	75	14.9	18	98	68.3	70	11.8	18.0	94.0	<0.001
**Body Mass Index (BMI. kg/m²)**	26.4	25.7	5.6	15.6	67.2	26.5	25.9	4.8	15.5	51.9	0.463
**Haemoglobin (g/dL)**	12.6	12.9	2.1	6.4	18.8	12.9	13.2	2.0	6.4	18.2	<0.001
**Mean Corpuscular Volume (MCV. fL)**	90.4	91	7.1	63.4	113.5	90.4	90.9	6.5	61.0	122.6	0.503
**Red blood cell Distribution Width (RDW. %)**	14.4	13.9	2.0	11.2	25.2	14.5	13.8	2.3	11.1	28.6	0.642
**Prothrombin time (PT. inr)**	1.1	1.04	0.4	0.8	5.1	1.1	1.01	0.2	0.8	4.4	<0.001
**Monocyte-to-Lymphocyte Ratio (MLR)**	0.3	0.3	0.3	0.07	2.8	0.4	0.3	0.2	0.0	2.13	0.069
**Neutrophil-to-Lymphocyte Ratio (NLR)**	3.2	2.3	3.5	0.4	47.7	2.9	2.2	2.8	0.5	43.1	0.122
**Platelet-to-lymphocyte Ratio (PLR)**	159.6	131.8	107.5	10.5	1528.9	158.9	132.3	103.1	35.2	1460.0	0.777

The table reports the distribution (number of patients n, median, min and max) of the key features, comparing patients who did not receive transfusion with those who received ≥1 unit. P-values were calculated according to the Wilcoxon-Mann-Whitney test.

### Comparison with logistic regression and decision curve analysis

Compared with conventional logistic regression analysis, our model achieved superior discriminatory performance, with an AUC of 0.80 (95% CI, 0.74–0.87) versus 0.78 (95% CI, 0.70–0.84) for logistic regression in the independent test set. These results are reported in Figure S2 of the Supplementary Material.

To further evaluate the potential clinical applicability of the model beyond discrimination metrics alone, we performed Decision Curve Analysis (DCA) [46,47]. DCA estimates the clinical net benefit of a prediction model across a range of threshold probabilities by balancing the benefit of correctly identifying true-positive cases against the harm associated with false-positive classifications.

The DCA showed that our model yielded a consistently higher net benefit than both the ‘treat-all’ and ‘treat-none’ strategies across a clinically relevant range of threshold probabilities (approximately 0.20–0.65). At lower threshold probabilities, the model performed similarly to the treat-all strategy, reflecting a preference for maximizing sensitivity. Conversely, at threshold probabilities above approximately 0.70, the net benefit progressively approached that of the treat-none strategy, indicating limited additional clinical value in this higher-threshold range. Details are reported in Figure S3 of the Supplementary Material.

## Discussion

This study developed and validated a machine-learning model based on CatBoost to predict the risk of intraoperative RBC transfusion. Using demographic, clinical, and laboratory data from more than 1,800 surgical patients, the model demonstrated stable and consistent performance across the training, validation, and test sets (Table 2 and Figure 3), with good discrimination (AUC 0.885, 0.841, and 0.805, respectively). Notably, the model achieved a consistently high NPV across all datasets (0.894 in training, 0.852 in validation, and 0.868 in the test set).

From a clinical perspective, the high NPV is particularly relevant because it allows reliable identification of patients at low risk of requiring intraoperative transfusion. Overall, the classifier demonstrates robust and clinically meaningful discrimination between patients who required transfusion (class 1) and those who did not (class 0), suggesting potential utility as a decision-support tool in surgical blood management.

The AUC remains fairly consistent, indicating only a modest loss of performance on unseen data. Sensitivity for the transfused class is high, supporting effective detection of transfused patients with comparatively few false negatives. Conversely, the specificity is moderate, suggesting a non-negligible false-positive rate (some patients who did not receive transfusions are predicted to have done so), particularly in the internal test set. This operating profile aligns with the negative and positive predictive values: NPV is consistently high (0.894/0.852/0.868), indicating that patients predicted as non-transfused are very likely to be truly non-transfused, whereas PPV is moderate (0.662/0.660/0.599), implying that a proportion of predicted transfusions will not be confirmed. These findings are consistent with the conclusions of earlier studies demonstrating the efficacy of gradient-boosting algorithms across a range of surgical contexts, including liver transplantation (AUC = 0.81) [34] and cardiac surgery (AUC ∼ 0.88) [35].

To further contextualize the predictive performance of the CatBoost model, we also compared it with a conventional logistic regression approach (Figure S2). Although both models showed good predictive ability, CatBoost achieved slightly superior performance, particularly in terms of discrimination and negative predictive value in the test set. In addition, DCA [47] (Figure S3) demonstrated that the model provided greater net clinical benefit than both the treat-all and treat-none strategies across a broad range of threshold probabilities, supporting its potential clinical utility in perioperative decision-making. This finding suggests that the model may support more effective clinical decision-making by improving patient stratification while minimizing unnecessary interventions.

Unlike linear regression algorithms, CatBoost also offers the advantage of directly integrating missing values and categorical variables without requiring extensive data transformation. This allowed us to develop a robust model with minimal data pre-processing (a step typically necessary for traditional linear algorithms), which may facilitate its implementation into clinical workflows.

The SHAP analysis was employed to identify clinically meaningful determinants of intraoperative transfusion risk. In line with the existing literature [14,48–50], the SHAP analysis identified the type of surgery [51], preoperative haemoglobin, RDW, BMI, PT and alteration of white blood cells-related parameters as the strongest predictors.

Among the types of surgery, orthopaedic was at the highest risk of intraoperative transfusion, with over 55% of patients receiving at least one RBC unit. The large majority of the orthopaedic cases (*n* = 572) were femur fracture (*n* = 322) or hip replacement (*n* = 156). These conditions typically affect the elderly [52], as confirmed by our data: the median age was 83 for femur fractures, and 75 for hip replacements (data not shown). Given the advanced age (often associated with comorbidities), these patient also had median haemoglobin levels typically lower than those of patients undergoing other surgical procedures: 12.4 g/dL for femur fractures and 12.0 g/dL for hip arthroplasties. This data contributes to lowering the median hemoglobin level of our cohort of non-oncological patients (Table 4).

Hip arthroplasty is known to be characterised by extensive tissue dissection, high vascularity and anatomical complexity, all of which contribute to increased intraoperative haemorrhage and, consequently, a higher likelihood of RBC transfusion. This finding is well supported by large observational studies and registry-based analyses reporting elevated transfusion rates in major orthopaedic procedures [2,53,54].

Similarly, urinary tract surgery (*n* = 266) showed a high risk of intraoperative transfusion (44% of these patients were transfused with at least 1 unit of RBC during surgery), with a median age of 72 years and 12.7 g/dl median haemoglobin (Supplementary Table 4). Here we collected all types of interventions that were not specifically classified as total or partial nephrectomies, prostatectomies, or bladder surgeries (detailed ICD-9 are shown in Supplementary Table 1). Patients undergoing urinary tract surgery had a median age of 72 years, with moderately low haemoglobin (median 12.7 g/dl) and high NL and PL ratios compared to the majority of other surgical groups.

Conversely, the negative correlations observed for lung, gynaecological, and bladder surgeries suggest that, within our cohort, these groups were associated with a lower inherent risk of transfusion. This observation is consistent with the literature describing relatively low transfusion rates for standard lung resections [29,55,56] and gynaecological procedures [57], particularly when performed electively.

Whilst bladder surgery has historically been associated with increased transfusion requirements [58,59], the negative correlation observed in this study may reflect cohort-specific factors (see Supplementary Table 5). These include the PBM strategies implemented in our hospital for both elective cancer [3] and non-cancer patients [12]. Indeed, recent evidence indicates that advances in surgical technique, anaesthesiologic management, and perioperative blood conservation protocols may mitigate transfusion risk [7,10,21,60,61].

Besides the type of surgery and haemoglobin levels, age was also found among the most important predicting features, probably due to impaired compensatory responses to blood loss [62] and increased morbidity and mortality after major surgery [63] in older adults. Indeed, patients of advanced age were found to have diminished physiological reserves, including reduced erythropoietic capacity and impaired responses to blood loss [63], potentially predisposing them both to anaemia and transfusion dependence in a surgical setting.

From the SHAP analysis BMI also emerged among the 10 most important features contributing to the predictions of the transfusion risk. In particular, low BMI indicated a higher intraoperative transfusion requirement. Despite obese patients (i.e. having a high BMI > 30) are at a higher risk of comorbidities that make them more likely to require orthopaedic surgery [64] among others, often experiencing complications post-surgery, their condition does not seem to affect intraoperative blood loss or blood transfusion rates [65]. This may be due to underlying disparities in nutritional status which may predispose low-weight patients to perioperative anaemia and impaired tolerance to blood loss [2,66,67].

Importantly, the presence of increased levels of leukocyte-derived ratios (MLR, NLR, PLR) and RDW among the top 10 features of the SHAP analysis suggests a role for preoperative systemic inflammation.

RDW is a measure of the variability in size of circulating RBC. Higher RDW (anisocytosis) reflect a higher variation in size, and this parameter has been used to differentiate between the causes of anemia. Indeed, anisocytosis occurs in anemia associated with iron, vitamin B12 or folate deficiency, as well as in pathological conditions that are associated to ineffective RBC production and/or increased destruction (such as myelodysplastic syndrome). In our cohort of surgical patients, RDW was significantly lower in males than in females, who were also likely to be more frequently transfused (38% of the total females have been transfused vs. 34% males).

Systemic inflammation indices such as MLR, NLR and PLR are emerging as prognostic markers in various pathological conditions [29,55,56]. A key practical advantage of these ratios is that they are derived from routine laboratory parameters, making them easy to obtain at essentially no additional cost. Risk prediction based on preoperative inflammatory ratios was first explored in oncology [68,69], and subsequently in cardiovascular medicine [70], before attracting growing interest in the perioperative setting. Given the acknowledged role of systemic inflammation in tumorigenesis and cancer progression [71], these markers have been extensively studied to predict surgical outcomes in oncological populations. NLR and other preoperative systemic indices have been associated with survival in head and neck cancer [72], pancreatic ductal adenocarcinoma [73] and colorectal cancer [74], while elevated preoperative NLR and PLR have recently been linked to poor prognosis and adverse surgical outcomes in renal cancer [75]. Indices combining platelet count with NLR have also been used to stratify mortality risk in elective surgical patients more generally [76]. Parallel evidence from cardiovascular and perioperative medicine reinforces this picture [70]: elevated preoperative NLR is associated with mortality and adverse outcomes after both cardiovascular [77] and non-cardiac surgery [78]. In elderly patients undergoing elective open cardiac surgery, poor postoperative prognosis was associated with higher preoperative NLR and PLR values [79].

Despite this substantial body of evidence, the relevance of these preoperative indices to elective surgery in general, and their correlation with blood transfusion has been only marginally explored [29,56,80].

One notable exception is the study by Park et al. [29], who demonstrated that the MLR was independently associated with intraoperative RBC transfusion during radical retropubic prostatectomy. In that patient cohort, comprising 1302 subjects, an MLR ≤ 4.3 was associated with a significant increase in the probability of transfusion (OR = 4.598, *p* < 0.001), with an AUC of 0.706 for predicting transfusion risk. Moreover, in population-based analyses, an elevated MLR has been associated with an augmented risk of anaemia [81], thereby indicating that a systemic inflammatory burden may contribute to haematological vulnerability.

Although elevated preoperative inflammatory ratios have been associated with adverse outcomes, previous studies suggest that these biomarkers generally provide only moderate predictive performance when used individually, limiting their clinical applicability as standalone predictors [82]. In this context, our machine learning model was able to effectively capture the contribution of inflammatory ratio levels in conjunction with other patient characteristics, thereby strengthening the model’s overall predictive performance for transfusion risk stratification. Beyond confirming known predictors such haemoglobin, our model is (to our knowledge) the first one highlighting the importance of derived inflammatory indices as relevant contributors to transfusion risk, providing additional biological and clinical insight into the relationship between systemic inflammation, surgical stress response, and transfusion requirements. Taken together, the presence of the MLR, NLR and PLR among the most important features of the SHAP analysis suggests the necessity of further investigation into the potential contribution of these indices to the improvement of transfusion risk in the context of PBM.

Comparing the distributions of the top features among oncologic and non-oncological patients, we found that haemoglobin was higher in the oncologic cohort and lower in non-oncological patients. Meanwhile, PT and age were significantly higher in non-cancer surgical patients (Table 4 and Supplementary Table 6). Reduced PT may be attributable to tumour procoagulant activity [81]. While higher haemoglobin levels in oncological patients compared to non-oncological patients may appear counterintuitive at first, this phenomenon can be attributed to the predominance of orthopaedic patients of advanced age among non-oncological patients of our cohort [62]. Systemic inflammatory markers such as MLR, NLR, and PLR have been widely investigated as cancer-associated biomarkers [69,73,75]: their levels are often influenced by tumor type, disease stage, treatment exposure, and patient-related factors [83,84]. Nevertheless, in our cohort, these inflammatory ratios did not show significant elevations in cancer patients compared with the overall study population. This finding suggests that, rather than acting as standalone discriminators of malignancy status, inflammatory ratios may capture patient-specific biological variability and contribute to transfusion risk prediction when integrated with other clinical variables.

This model provides a multivariate foundation for pre-operative risk stratification that extends beyond haemoglobin levels alone. Our machine learning tool was specifically designed to handle heterogeneous, real-world clinical data. The model was developed on a broad and diverse surgical population, including multiple types of major and oncologic procedures. Furthermore, the use of CatBoost required only minimal data pre-processing, setting our work apart from previous studies that focused on single surgical domains. This approach supports the development of a more generalizable tool that can be easily translated into clinical practice. From a biological and clinical perspective, the identified features and inflammatory ratios, together with the wide applicability, represent a step towards a personalised approach to the preoperative management of surgical patients. From an operational perspective, the emergence of these inflammatory indices suggests that future preoperative management of surgical patients should consider coupling anti-inflammatory therapies with the management of anaemia.

In conclusion, the implementation of this system has the potential to streamline the management of preoperative anaemia correction, optimise blood ordering processes, and enhance clinical decision-making.

### Limitation and future perspective

Several limitations of this study should be acknowledged. The retrospective, single-center design may limit the generalizability of our findings, as patient case mix, perioperative workflows, transfusion thresholds, and surgical and anaesthetic practices can vary substantially across institutions. Such inter-center heterogeneity may influence model performance if applied in different clinical settings. In addition, although SHAP analysis significantly improves model interpretability, the underlying decision-making process of the machine learning framework remains inherently complex, which may represent a barrier to widespread clinical adoption. Missing data, although handled natively by CatBoost, may have introduced residual bias, particularly if data were not missing completely at random.

Another limitation is the absence of an explicit evaluation of the contribution of individual surgeons and anesthesiologists to transfusion prediction. Previous studies have demonstrated significant inter-surgeon [51,85–87] and inter-anesthesiologist variability in transfusion practices [88], highlighting the influence of operator-dependent factors on perioperative blood utilization.

However, as these variables are highly institution-specific, their inclusion would require center-tailored modelling approaches to preserve generalizability. Nonetheless, the role of the intraoperative care team in shaping transfusion decisions warrants further investigation in future studies [54].

To enhance the robustness and clinical utility of this predictive framework, future adjustments should be encouraged. External validation across diverse surgical settings and patient populations is essential to confirm model performance and transportability. Prospective implementation studies are also needed to assess whether real-time integration of the model into perioperative workflows and electronic health record–based clinical decision support systems can effectively reduce transfusion rates, improve patient outcomes, and generate cost savings. Finally, embedding the model within structured PBM programmes, supported by user-friendly digital interfaces and intuitive SHAP-based visualisations, may facilitate clinician engagement and enable transparent, real-time assessment of transfusion risk at the point of care.

## Supplementary Material

Supplemental Material

## Data Availability

The datasets and the scripts used for model development and evaluation are available from the corresponding author upon reasonable request and in accordance with institutional and ethical regulations.
