# Supplementary material for: Machine learning-based prediction of intraoperative blood transfusion in major surgery: exploiting clinical variables and systemic inflammatory indices
Source: Ann Med. 2026 Aug 10;58(1):2707807. doi: 10.1080/07853890.2026.2707807 (PMC13463459; doi:10.1080/07853890.2026.2707807)
Supplement: Supplementary file 1 [file IANN_A_2707807_SM1619.docx]

**Supplementary material**

**Supplementary table 1.** ICD-9 codes for the groups of surgeries considered:

| **Type of Surgery** | **ICD-9** |
| --- | --- |
| Digestive System Surgery | 41.5, 41.5, 42.0, 42.42, 43.0, 43.6, 43.99, 44, 44.31, 44.43, 45.3, 45.62, 45.76, 45.8, 46, 48, 48.5, 48.69, 48.8, 51.0, 52.6, 52.7 |
| Kidney | 55-xx, 55.4, 55.5, 55.51 |
| Endocrine Surgery | 06.4, 07.2 |
| Gyneccologic Surgery | 6, 7, 68.29, 68.4, 68.9 |
| Prostate Surgery | 60.21, 65, 65.98, 68 |
| Lung Surgery | 32.4, 32.41, 32.5, 32.9, 33.99, 34, 34.2, 34.9, 60.29, 60.5 |
| Vascular Surgery | 38, 38.14, 38.29, 38.44, 38.45, 39, 39.25 |
| Bladder Surgery | 57.49, 57.71 |
| Urinary tract Surgery | 56.0, 56.9, 57.33, 59, 59.99 |
| Epatectomy | 50.22, 50.4 |
| Orthopedics | 70, 71, 72, 77, 78.60, 79.10, 79.15, 80, 80.26, 81.16, 81.51, 81.53, 81.54, 81.55, 81.80, 84.5, 800-829 |
| Others | 54.0, 99.9 |

**Figure S1.** Model calibration assessment. The figure shows the calibration plots for training (left panel), validation (central panel), and test dataset (right panel).

**Supplementary Table 2.** Calibration metrics:

| *Metric* | *Training* | *Validation* | *Test* |
| --- | --- | --- | --- |
| Brier score | 0.14 | 0.16 | 0.18 |
| Log loss | 0.44 | 0.50 | 0.54 |
| Calibration intercept | -0.71 | -0.49 | -0.56 |
| Calibration slope | 1.58 | 1.19 | 1.05 |
| Expected calibration error | 0.12 | 0.09 | 0.11 |

Brier Score, Log Loss (Binary Cross-Entropy), Calibration Intercept, Calibration Slope, and Expected Calibration Error (ECE). The Brief Score measures the accuracy of probabilistic predictions, and lower scores are better. Log Loss (Binary Cross-Entropy) evaluates the probabilistic prediction, and lower values are better. Calibration intercept is 0 for an ideal model: negative values indicate a model’s overconfidence, while positive intercepts suggest underconfidence. In an ideal model, the Calibration Slope is 1; values greater than 1 indicate underconfidence, while values less than 1 indicate overconfidence. ECE quantifies the difference between a model's expected and actual accuracy across different confidence bins. A lower ECE indicated better calibration.

Brier scores show a slight increase from the training to the validation and test sets, indicating very slightly lower accuracy on new data. Similarly, the Log Loss increases in the same way. The train’s Calibration slope is 1.58, indicating a sort of underconfidence. However, it improves on validation and test sets. This is a positive sign indicating that our model’s predicted probabilities are becoming better aligned with the actual outcome on new data. The calibration slope is close to 1, and it improves moving on the test set. This is a strong indicator that the spread of our model's probabilities is becoming more accurate as it generalizes. Moreover, ECE’s values indicate that the calibration error is not drastically increasing on unseen data.

**Performance of the logistic regression model**

**A**

**B**

**C**

**Figure S2.** Logistic regression model’s performance. AUCs (left panels) and confusion matrices (right panels) for training (A), validating (B), and test (C) dataset

**Net‑benefit analysis**

Decision curve analysis indicates whether using the model to guide clinical decisions yields greater benefit than default strategies such as treating all patients or treating none.

**Figure S3.** Decision Curve Analysis for training (upper left), validating (upper right), and test dataset (lower panel).

In our results, the model’s curves lye above both the treat-all and treat-none strategies for all the datasets, indicating a positive net benefit. In particular, for the test set, this advantage is observed across a broad range of threshold probabilities (approximately 0.15 to 0.65–0.70), suggesting potential clinical utility within this decision interval. At very low thresholds, the model performed similarly to the treat-all strategy, whereas at higher thresholds (above ~0.70), the net benefit approached that of treating none.

**Supplementary Table 3.** Distribution of the predictors identified by the CatBoost model according to sex:

|  | **Female (n = 835)** | | | | | **Male (n = 1023)** | | | | |  |
| --- | --- | --- | --- | --- | --- | --- | --- | --- | --- | --- | --- |
|  | **transfused n = 317** | | | | | **transfused n = 347** | | | | |  |
| **Predictor** | **mean** | **median** | **SD** | **min** | **max** | **mean** | **median** | **SD** | **min** | **max** | **p-value** |
| **Age (years)** | 70.768 | 74 | 14.571 | 18 | 98 | 69.89 | 72 | 12.613 | 18 | 98 | 0.008 |
| **Body Mass Index (BMI. kg/m²)** | 26.253 | 25 | 6.217 | 15.6 | 67.2 | 26.691 | 26.1 | 4.203 | 15.5 | 44.1 | <0.001 |
| **Haemoglobin (g/dL)** | 12.152 | 12.4 | 1.834 | 6.4 | 16.9 | 13.348 | 13.6 | 2.107 | 6.4 | 18.8 | <0.001 |
| **Mean Corpuscular Volume (MCV. fL)** | 89.654 | 90.4 | 7.312 | 61 | 122.6 | 91.038 | 91.2 | 6.324 | 63.1 | 121.3 | <0.001 |
| **Red blood cell Distribution Width (RDW. %)** | 14.651 | 14 | 2.315 | 11.4 | 26.6 | 14.306 | 13.8 | 2.044 | 11.1 | 28.6 | 0.001 |
| **Prothrombin time (PT. inr)** | 1.102 | 1.01 | 0.324 | 0.8 | 4.38 | 1.082 | 1.02 | 0.291 | 0.81 | 5.1 | 0.497 |
| **Monocyte-to-Lymphocyte Ratio (MLR)** | 0.369 | 0.296 | 0.259 | 0.0253 | 2.82 | 0.39 | 0.317 | 0.254 | 0.0709 | 2.58 | <0.001 |
| **Neutrophil-to-Lymphocyte Ratio (NLR)** | 3.166 | 2.278 | 3.637 | 0.4393 | 47.75 | 3.06 | 2.296 | 2.792 | 0.5185 | 25.83 | 0.814 |
| **Platelet-to-lymphocyte Ratio (PLR)** | 177.634 | 145.833 | 121.254 | 33.8422 | 1528.95 | 144.318 | 125.391 | 87.4 | 10.4816 | 1460 | <0.001 |

**Supplementary Table 4.** Distribution of the predictors identified by the CatBoost model according to the group of surgery:

|  |  | **Urinary tract surgery** | **Liver surgery** | **Digestive surgery** | **Kidney surgery** | **Prostate surgery** | **Orthopedic surgery** | **Endocrine surgery** | **Vascular surgery** | **Bladder surgery** | **Abdominal surgery** | **Trauma** | **Gynecologic surgery** | **Lung surgery** | **Other** | **Not specified** |
| --- | --- | --- | --- | --- | --- | --- | --- | --- | --- | --- | --- | --- | --- | --- | --- | --- |
|  | **n** | 266.0 | 21.0 | 418.0 | 160.0 | 83.0 | 572.0 | 40.0 | 89.0 | 34.0 | 28.0 | 1.0 | 69.0 | 60.0 | 8.0 | 9.0 |
| **Age (years)** | **mean** | 68.9 | 72.2 | 66.1 | 67.5 | 68.4 | 76.9 | 53.0 | 74.5 | 75.7 | 59.2 | 63.0 | 64.3 | 67.6 | 76.0 | 57.0 |
|  | **median** | 72.0 | 74.0 | 67.0 | 68.5 | 68.0 | 78.0 | 55.0 | 76.0 | 76.0 | 65.0 | 63.0 | 65.0 | 71.0 | 82.0 | 52.5 |
|  | **SD** | 14.1 | 7.9 | 13.3 | 11.6 | 7.4 | 12.8 | 11.4 | 8.8 | 7.4 | 12.9 | NaN | 12.6 | 8.7 | 10.4 | 17.8 |
|  | **min** | 20.0 | 58.0 | 17.0 | 32.0 | 47.0 | 8.0 | 32.0 | 45.0 | 42.0 | 33.0 | 63.0 | 32.0 | 48.0 | 64.0 | 42.0 |
|  | **max** | 94.0 | 84.0 | 90.0 | 87.0 | 89.0 | 98.0 | 76.0 | 90.0 | 89.0 | 80.0 | 63.0 | 90.0 | 79.0 | 82.0 | 81.0 |
| **Haemoglobin (g/dl)** | **mean** | 12.5 | 13.2 | 12.7 | 13.7 | 14.9 | 12.5 | 13.6 | 13.2 | 12.1 | 10.8 | 13.0 | 12.2 | 13.7 | 11.3 | 12.3 |
|  | **median** | 12.7 | 13.2 | 12.9 | 13.9 | 14.9 | 12.7 | 13.4 | 13.7 | 11.9 | 10.7 | 13.0 | 12.4 | 13.8 | 11.0 | 12.3 |
|  | **SD** | 2.4 | 1.5 | 2.1 | 1.7 | 1.2 | 1.9 | 1.9 | 2.0 | 2.2 | 2.1 | NaN | 16.5 | 15.8 | 3.6 | 2.8 |
|  | **min** | 7.0 | 10.7 | 6.4 | 8.2 | 12.1 | 7.3 | 7.6 | 7.7 | 7.6 | 7.5 | 13.0 | 7.6 | 9.4 | 7.9 | 9.0 |
|  | **max** | 18.8 | 16.5 | 17.3 | 16.9 | 18.1 | 16.9 | 17.8 | 17.2 | 15.9 | 15.2 | 13.0 | 15.8 | 18.2 | 15.0 | 15.5 |
| **BMI** | **mean** | 26.5 | 28.1 | 26.7 | 26.6 | 26.7 | 26.0 | 29.1 | 25.3 | 25.8 | 29.5 | 32.9 | 27.3 | 26.3 | 27.8 | 23.1 |
|  | **median** | 26.1 | 27.5 | 25.9 | 25.9 | 26.0 | 25.5 | 29.3 | 25.1 | 25.0 | 30.6 | 32.9 | 25.4 | 25.5 | 30.5 | 21.0 |
|  | **SD** | 4.5 | 5.7 | 5.8 | 5.3 | 3.1 | 5.2 | 4.8 | 4.3 | 3.9 | 6.3 | NaN | 6.4 | 4.0 | 4.6 | 4.5 |
|  | **min** | 16.2 | 17.7 | 15.6 | 15.5 | 20.8 | 15.8 | 18.5 | 16.6 | 19.2 | 18.4 | 32.9 | 17.8 | 19.1 | 22.5 | 20.6 |
|  | **max** | 41.5 | 40.6 | 67.2 | 51.9 | 36.4 | 44.9 | 39.5 | 48.9 | 34.4 | 43.8 | 32.9 | 49.5 | 39.8 | 30.5 | 29.8 |
| **MCV (fl)** | **mean** | 90.5 | 92.8 | 88.5 | 90.9 | 91.8 | 90.7 | 89.5 | 92.9 | 91.7 | 87.8 | 92.4 | 91.5 | 91.6 | 92.0 | 89.3 |
|  | **median** | 90.9 | 91.0 | 89.8 | 91.2 | 91.3 | 91.4 | 89.8 | 92.5 | 90.9 | 87.7 | 92.4 | 92.0 | 92.2 | 91.9 | 90.3 |
|  | **SD** | 6.1 | 9.2 | 7.2 | 4.7 | 6.0 | 7.4 | 3.6 | 6.4 | 6.8 | 7.8 | NaN | 6.3 | 6.4 | 3.2 | 3.7 |
|  | **min** | 63.1 | 75.3 | 61.0 | 75.3 | 68.7 | 63.4 | 81.7 | 73.0 | 75.1 | 67.0 | 92.4 | 72.0 | 74.0 | 88.9 | 84.4 |
|  | **max** | 106.9 | 122.6 | 112.5 | 102.3 | 121.3 | 113.5 | 97.4 | 113.1 | 108.5 | 103.7 | 92.4 | 106.7 | 113.6 | 95.3 | 92.2 |
| **RDW (%)** | **mean** | 14.4 | 14.4 | 15.1 | 13.7 | 13.3 | 14.5 | 13.3 | 14.3 | 14.9 | 15.5 | 13.6 | 14.5 | 13.7 | 14.4 | 13.8 |
|  | **median** | 13.8 | 14.0 | 14.2 | 13.4 | 13.1 | 14.1 | 13.2 | 13.7 | 14.6 | 15.7 | 13.6 | 13.5 | 13.3 | 14.2 | 13.9 |
|  | **SD** | 21.3 | 18.7 | 27.4 | 13.4 | 0.9 | 19.4 | 10.0 | 18.7 | 19.3 | 21.3 | NaN | 28.2 | 15.1 | 13.7 | 11.6 |
|  | **min** | 11.2 | 12.4 | 11.8 | 11.6 | 11.1 | 11.9 | 11.9 | 12.1 | 11.9 | 12.3 | 13.6 | 11.4 | 11.7 | 13.2 | 12.4 |
|  | **max** | 26.6 | 19.1 | 28.6 | 18.0 | 16.4 | 25.2 | 17.0 | 21.2 | 20.0 | 20.7 | 13.6 | 24.8 | 21.9 | 15.9 | 14.9 |
| **PT (inr)** | **mean** | 1.1 | 1.1 | 1.0 | 1.0 | 1.0 | 1.2 | 1.0 | 1.1 | 1.1 | 1.1 | 1.0 | 1.0 | 1.0 | 1.1 | 1.0 |
|  | **median** | 1.0 | 1.1 | 1.0 | 1.0 | 1.0 | 1.1 | 1.0 | 1.0 | 1.1 | 1.1 | 1.0 | 1.0 | 1.0 | 1.1 | 1.0 |
|  | **SD** | 0.4 | 0.1 | 0.2 | 0.2 | 0.3 | 0.4 | 0.1 | 0.4 | 0.1 | 0.2 | NaN | 0.1 | 0.1 | 0.1 | 0.1 |
|  | **min** | 0.8 | 0.8 | 0.8 | 0.8 | 0.8 | 0.9 | 0.9 | 0.9 | 0.9 | 0.9 | 1000.0 | 0.9 | 0.9 | 1050.0 | 0.9 |
|  | **max** | 5.1 | 1.3 | 4.4 | 2.8 | 3.2 | 3.3 | 1.3 | 3.4 | 1.3 | 1.5 | 1.0 | 1.2 | 1.2 | 1.3 | 1.0 |
| **ML ratio** | **mean** | 0.4 | 0.3 | 0.4 | 0.3 | 0.3 | 0.4 | 0.3 | 0.4 | 0.4 | 0.6 | 0.3 | 0.3 | 0.4 | 0.7 | 0.2 |
|  | **median** | 0.3 | 0.3 | 0.3 | 0.3 | 0.3 | 0.3 | 0.3 | 0.3 | 0.4 | 0.4 | 0.3 | 0.3 | 0.3 | 0.5 | 0.3 |
|  | **SD** | 0.3 | 0.2 | 0.3 | 0.2 | 0.1 | 0.3 | 0.1 | 0.2 | 0.2 | 0.4 | NaN | 0.1 | 0.2 | 0.6 | 0.0 |
|  | **min** | 0.1 | 0.1 | 0.0 | 0.1 | 0.1 | 0.1 | 0.1 | 0.2 | 0.1 | 0.1 | 0.3 | 0.1 | 0.1 | 0.2 | 0.2 |
|  | **max** | 2.1 | 0.7 | 2.1 | 1.7 | 0.6 | 2.8 | 0.5 | 1.2 | 1.0 | 2.0 | 0.3 | 0.7 | 1.0 | 1.3 | 0.3 |
| **NL ratio** | **mean** | 3.5 | 2.1 | 3.3 | 2.5 | 2.1 | 3.3 | 2.4 | 2.8 | 3.8 | 5.1 | 2.6 | 2.5 | 2.4 | 7.1 | 2.3 |
|  | **median** | 2.5 | 2.0 | 2.2 | 2.0 | 1.8 | 2.4 | 2.2 | 2.3 | 2.8 | 3.2 | 2.6 | 2.2 | 2.0 | 2.0 | 2.3 |
|  | **SD** | 3.6 | 1.0 | 3.3 | 1.6 | 1.0 | 3.6 | 1.2 | 2.4 | 3.8 | 5.1 | NaN | 1.7 | 1.4 | 9.3 | 1.1 |
|  | **min** | 0.9 | 0.8 | 0.5 | 0.8 | 0.7 | 0.4 | 0.9 | 1.0 | 0.7 | 0.7 | 2.6 | 0.8 | 0.7 | 1.5 | 1.1 |
|  | **max** | 43.1 | 4.4 | 32.4 | 11.8 | 4.9 | 47.8 | 5.4 | 20.3 | 21.7 | 16.8 | 2.6 | 13.2 | 6.9 | 17.8 | 3.7 |
| **PT ratio** | **mean** | 159.0 | 127.9 | 173.7 | 140.1 | 116.5 | 166.6 | 151.5 | 128.7 | 167.5 | 176.9 | 145.5 | 161.9 | 142.1 | 239.1 | 148.7 |
|  | **median** | 135.3 | 108.5 | 136.4 | 130.0 | 110.5 | 137.8 | 144.0 | 110.9 | 155.6 | 149.5 | 145.5 | 136.2 | 129.9 | 109.4 | 121.1 |
|  | **SD** | 102.2 | 81.0 | 130.0 | 55.3 | 38.6 | 115.7 | 65.2 | 61.0 | 79.2 | 108.1 | NaN | 81.9 | 59.6 | 229.4 | 85.3 |
|  | **min** | 35.2 | 47.8 | 10.5 | 47.9 | 36.5 | 33.8 | 58.2 | 33.1 | 38.0 | 37.1 | 145.5 | 39.2 | 57.2 | 103.9 | 83.3 |
|  | **max** | 1045.5 | 354.2 | 1460.0 | 403.9 | 243.4 | 1528.9 | 313.4 | 310.7 | 367.0 | 559.6 | 145.5 | 587.7 | 332.2 | 503.9 | 269.3 |

**Supplementary Table 5.** Number of transfused and non-transfused patients according to the group of surgery:

| **Group of surgery** | **n = 1858** | **Transfused** | **Not transfused** |
| --- | --- | --- | --- |
|  |  | **n = 664** | **n = 1194** |
| **Urinary tract surgery** | 266 | 118 | 148 |
| **Liver surgery** | 21 | 4 | 17 |
| **Digestive surgery** | 418 | 99 | 319 |
| **Kidney surgery** | 160 | 34 | 126 |
| **Prostate surgery** | 83 | 5 | 78 |
| **Orthopedic surgery** | 572 | 329 | 243 |
| **Endocrine surgery** | 40 | 3 | 37 |
| **Vascular surgery** | 89 | 20 | 69 |
| **Bladder surgery** | 34 | 11 | 23 |
| **Abdominal surgery** | 28 | 13 | 15 |
| **Trauma** | 1 | 0 | 1 |
| **Gynecologic surgery** | 69 | 17 | 52 |
| **Lung surgery** | 60 | 5 | 55 |
| **Other** | 8 | 3 | 5 |
| **Not specified** | 9 | 3 | 6 |

**Supplementary Table 6.** Distribution of the predictors identified by the CatBoost model according to cancer diagnosis:

|  | **Non oncologic (n = 900)** | | | | | **Oncologic (n = 958)** | | | | |  | |
| --- | --- | --- | --- | --- | --- | --- | --- | --- | --- | --- | --- | --- |
|  | **transfused n = 330** | | | | | **transfused n = 334** | | | | |  | |
| **Predictor** |  | **median** | **SD** | **min** | **max** | **mean** | **median** | **SD** | **min** | **max** | **p-value** |  |
| **Age (years)** | 72.3 | 75 | 14.9 | 8.0 | 98.0 | 68.3 | 70 | 11.789 | 17 | 94 | <0.001 |  |
| **Body Mass Index (BMI. kg/m²)** | 26.5 | 25.7 | 5.6 | 15.6 | 67.2 | 26.5 | 25.9 | 4.848 | 15.5 | 51.9 | 0.463 |  |
| **Haemoglobin (g/dL)** | 12.6 | 12.9 | 2.1 | 6.4 | 18.8 | 12.9 | 13.2 | 2.037 | 6.4 | 18.2 | <0.001 |  |
| **Mean Corpuscular Volume (MCV. fL)** | 90.4 | 91 | 7.0 | 63.4 | 113.5 | 90.4 | 90.9 | 6.555 | 61 | 122.6 | 0.503 |  |
| **Red blood cell Distribution Width (RDW. %)** | 14.4 | 13.9 | 2.0 | 11.2 | 25.2 | 14.5 | 13.8 | 2.31 | 11.1 | 28.6 | 0.642 |  |
| **Prothrombin time (PT. inr)** | 1.1 | 1.0 | 0.4 | 0.8 | 5.1 | 1.0 | 1.01 | 0.239 | 0.81 | 4.38 | <0.001 |  |
| **Monocyte-to-Lymphocyte Ratio (MLR)** | 0.4 | 0.3 | 0.3 | 0.07 | 2.8 | 0.4 | 0.301 | 0.248 | 0.0253 | 2.13 | 0.069 |  |
| **Neutrophil-to-Lymphocyte Ratio (NLR)** | 3.2 | 2.3 | 3.5 | 0.4 | 47.7 | 2.9 | 2.188 | 2.893 | 0.5468 | 43.11 | 0.122 |  |
| **Platelet-to-lymphocyte Ratio (PLR)** | 159.6 | 131.8 | 107.5 | 10.5 | 1528.9 | 158.9 | 132.37 | 103.087 | 35.2239 | 1460 | 0.777 |  |
